# Food protein-induced eosinophilic enteritis with intestinal stricture in a neonate: a case report and review of the literature

**DOI:** 10.1093/jscr/rjy170

**Published:** 2018-07-21

**Authors:** Yukihiro Tatekawa

**Affiliations:** Department of Pediatric Surgery, Saku Central Hospital Advanced Care Center, 3400-28, Nakagomi, Saku-shi, Nagano, Japan

## Abstract

The case of a 21-day-old boy with eosinophilic enteritis with intestinal stricture due to a food protein-induced allergy is reported herein. For the first 4 days of life, he was both breast-fed and formula-fed, after which he was exclusively breast-fed. At the age of 24 days, he underwent laparotomy to investigate the possibility of intestinal obstruction for bilious vomiting, and an ileal stricture was detected and resected. Pathologic analysis showed the presence of eosinophil accumulation in the lesions presented more than 20 eosinophils per high-power field and the eosinophils were oriented towards the epithelium and diffusely distributed throughout the tissue, but the margins of resection showed a few infiltration of eosinophiles. Allergen-specific lymphocyte stimulation testing showed a markedly increased lymphocyte response to lactoferrin. He was finally diagnosed as eosinophilic enteritis with intestinal stricture due to a food protein-induced allergy. The patient remained asymptomatic during a follow-up period of 12 months.

## INTRODUCTION

Gastrointestinal food-induced allergic disorders that are not mediated by immunoglobulin E (non-IgE-GI-FAs) account for an unknown proportion of food allergies and include food protein-induced enterocolitis syndrome, food protein-induced allergic proctocolitis, and food protein-induced enteropathy. The non-IgE-GI-FAs have many overlapping clinical and histologic features, both among themselves and between these entities and the eosinophilic gastroenteropathies [1]. In the neonatal period, non-IgE-GI-FAs present with diarrhea, vomiting, abdominal distension, bloody stools and failure to thrive. These symptoms are usually attributed to a variety of other neonatal conditions such as sepsis or surgical gastrointestinal disease, e.g. gastrointestinal obstruction or intestinal perforation [2].

The eosinophilic gastroenteropathies—gastroenteritis and enteritis—are disorders characterized by infiltration of eosinophils in the gastrointestinal tract. Food allergies can be a triggering factor, especially in children. Accumulation of eosinophils in the gut due to food-induced allergy is regulated through a complex molecular network involving type 2 helper cells, cytokines and chemokines, which cause the severe tissue damage characteristic of eosinophilic gastroenteritis [3].

## CASE REPORT

**Figure 1:**
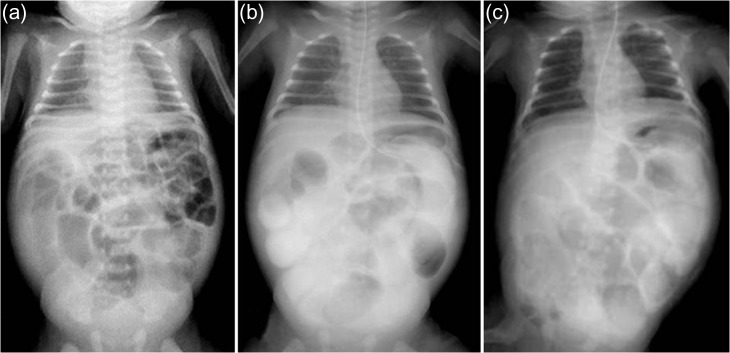
Abdominal radiography. (**a**) Admission films show partially dilated loops of bowel with intestinal gas (age 20 days). (**b** and **c**) Time progression radiography. Five hours after injecting contrast through a gastric tube (**b**, age 23 days) and 16 h after injecting contrast medium (**c**, age 24 days); dilated loops of bowel with intestinal gas are visible, with residual contrast medium.

**Figure 2:**
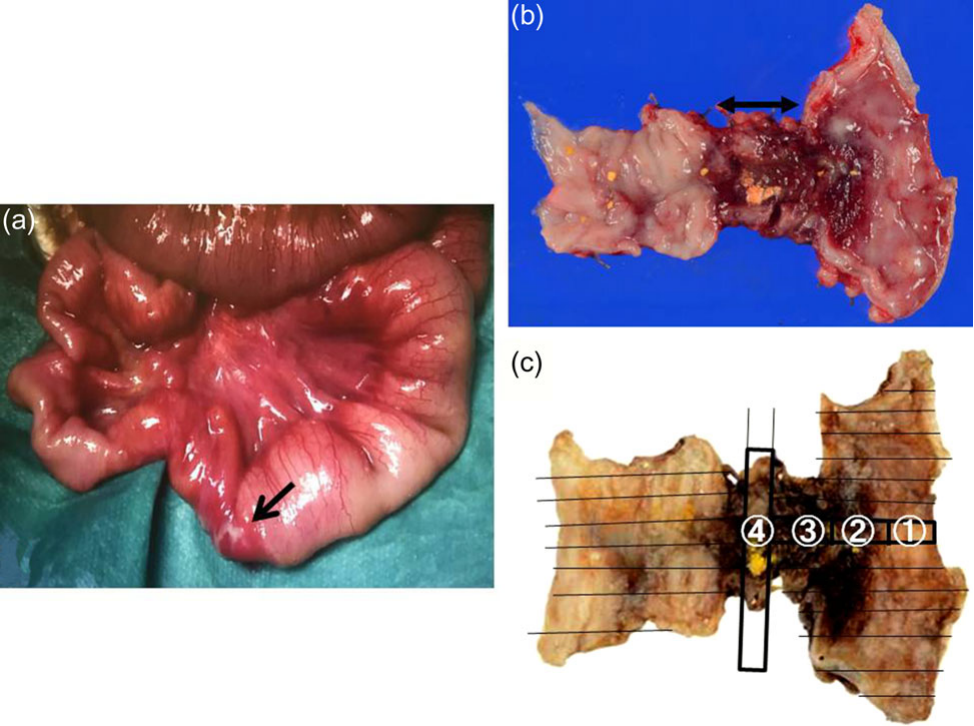
Intraoperative findings, resected specimen and formalin fixation of the specimen with slice. (**a**) A change in caliber with a stricture in the ileum is visible about 10 cm proximal to the ileocecal valve (black arrow indicates the stricture). (**b**) In the resected specimen, the lesion is noted to be 1.5 cm in length, featuring a stricture and ulceration. (**c**) Formalin fixation of the specimen is cut with slices.

**Figure 3:**
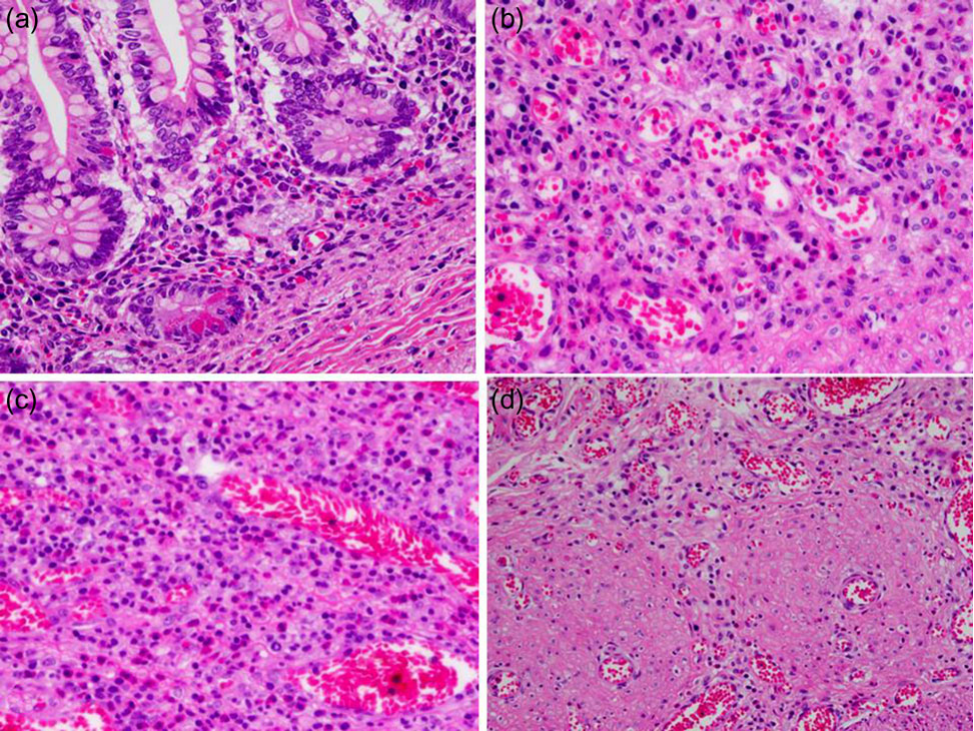
Microscopic pathology. (**a**) The margins of resection show a few infiltration of eosinophil (×400; ① in Fig. 2c). (**b**, **c**) The presence of eosinophil accumulation in the lesions presents more than 20 eosinophils per high-power field (HPF) (b ×400; ② in Fig. 2c, c ×400; ③ in Fig. 2c). (**d**) In the stenotic portion of the specimen, the layers between the mucosa and the muscularis propria are notably absent, and granulation and fibrotic tissues are found (d ×400; ④ in Fig. 2c).

A 21-day-old boy with vomiting, abdominal distention and feeding intolerance presented to our institution. He was born at 36 weeks and 6 days of gestation, weighing 2220 g, with Apgar scores of 8 at 1 min and 8 at 5 min. For the first 4 days of life, he was both breast- and formula-fed. After hospital discharge, he was exclusively breast-fed. He experienced occasional vomiting until 19 days of age, when he developed frequent vomiting. When he was 20 days old, he was taken to the doctor for several days of watery stools, a single episode of bilious vomiting, and feeding intolerance. He was admitted to a local hospital at a weight of 2685 g. Abdominal radiography showed partially dilated loops of bowel with intestinal gas (Fig. 1a), and a gastric tube was inserted for frequent vomiting. At the age of 21 days, he was transferred to our hospital for further examination. A gastrointestinal X-ray series and an enema revealed gastric volvulus and gastroesophageal reflux, without intestinal malrotation or a change in intestinal caliber. We admitted the patient for observation. The following day, an abdominal radiograph showed complete passage of contrast, which indicated the absence of intestinal atresia or obstruction. However, the volume of bile discharged through the gastric tube was gradually increasing, and he had little passage of feces, even with a glycerin enema. We decided to re-evaluate for intestinal obstruction and injected contrast into the gastric tube. Follow-up abdominal radiography showed obvious intestinal dilation with gas and retention of the contrast medium (Fig. 1b and c). Based on his clinical course and radiological findings, we suspected distal intestinal obstruction. When the patient was 24 days of age, we performed laparotomy, which revealed a caliber change in the ileum with a stricture ~10 cm proximal to the ileocecal valve (Fig. 2a). A 6-cm length of bowel around the stricture site was resected, and an end-to-end anastomosis was performed. On gross findings of the resected specimen, the lesion was noted to be 1.5 cm in length, featuring a stricture and erosion/ulceration (Fig. 2b and c). On microscopic examination, the margins of resection showed <20 eosinophils per high-power field (HPF) (×400) (Fig. 3a). However, mucosal eosinophilia was recognized in distant position from the ulcer (Fig. 3b and c)*.* The presence of eosinophil accumulation in the lesions presented more than 20 eosinophils per high-power field (HPF) (×400). The eosinophils were oriented towards the epithelium and diffusely distributed throughout the tissue. In the stenotic portion of the specimen, the layers between the mucosa and the muscularis propria were notably absent, and granulation and fibrotic tissues were found (Fig. 3d).

Based on these findings, the suspected diagnosis was eosinophilic enteritis. However, laboratory data on admission showed no hypereosinophilia (white blood cell count, 4800/μL with 1.4% eosinophils). Serum allergy investigation revealed no remarkable elevation in non-specific IgE, although the C-reactive protein level was slightly elevated (Table 1). Nevertheless, we strongly suspected a food protein-induced allergy to cow’s milk or breast milk. An allergen-specific lymphocyte stimulation test was performed for kappa (κ)-casein, lactoferrin and human alpha (α)-lactalbumin (outsourced to Bio Medical Laboratories [BML, Inc, Tokyo, Japan]) [4]. The lymphocyte response to lactoferrin was markedly increased (7489 counts per minute; stimulation index, 10.11; cutoff index, 3.9) (Table 1). He was finally diagnosed as eosinophilic enteritis with intestinal stricture caused by an allergy to either cow’s milk or breast milk. After surgery, the patient was fed with a pediatric elemental formula. His subsequent hospital course was uneventful, and he was discharged with no symptoms and good weight gain on the 19th postoperative day. At 5 months of age, he was able to eat baby food with no recurrence of symptoms. At 1 year of age, favorable growth and development were confirmed.

**Table 1:** Results of laboratory examination on admission and allergen-specific lymphocyte stimulation test after surgery

Hospital laboratory evaluation			
Admission laboratory data			
WBC	4.8 × 10^3^	(/μL)	(4.0–9.0)
Neut	24.5	(%)	(37.0–73.0)
Eosino	1.4	(%)	(0.5–11.0)
Baso	0.2	(%)	(0.5–11.0)
Lymph	47.5	(%)	(20.0–55.0)
Mono	26.4	(%)	(2.5–10.0)
RBC	392 × 10^4^	(/μL)	(420–540)
Hb	13.0	(g/dL)	(13.0–18.0)
Ht	36.6	(%)	(39.0–52.0)
Plts	45.4 × 10^4^	(/μL)	(15.0–35.0)
CRP	1.37	(mg/dL)	(<0.3)
Non-specific IgE	<5	(IU/mL)	(<20; under the age of 1 year)

Antigen	Count value (cpm)	SI	RR	Cutoff index
ALST after surgery				
κ-Casein	532	0.72	1.58	0.5
Lactoferrin	7489	10.11	2.62	3.9
Human α-lactalbumin	763	1.03	2.27	0.5

Laboratory investigation on admission shows no hypereosinophilia. Serum allergy investigation reveals no remarkable elevation in nonspecific immunoglobulin E. The allergen-specific lymphocyte stimulation test shows a markedly increased lymphocyte response to lactoferrin. cpm = counts per minute; SI = stimulation index; RR = reference ranges; SI = (mean cpm with treatment)/(mean cpm without treatment); cutoff index = SI/RR.

## DISCUSSION

Cow’s milk protein allergy is the most common food allergy in infants. Most neonatal milk allergies fall into the category of non-IgE-GI-FAs. Cow’s milk-induced enterocolitis has been described as presenting as early as the first week of life in several reports. This very early form of allergic enterocolitis suggests intrauterine sensitization caused by cow’s milk antigen in either the amniotic fluid or the placental blood supply; this antigen elicits an allergic response in the fetus [1]. There are recent reports of food protein-induced enterocolitis syndrome in exclusively breast-fed infants, suggesting that cow’s milk proteins excreted in breast milk are capable of causing an allergic reaction [5, 6]. In a report of an exclusively breast-fed neonate with cow’s milk-induced colitis, a rectal biopsy showed marked eosinophilic infiltration [7]. Another report of eosinophilic enteritis due to cow’s milk allergy describes eosinophilic infiltration in a specimen obtained at ileostomy after feeding with cow’s milk [8]. Accumulation of eosinophils in the gut is a common feature in food-induced allergies.

In eosinophilic gastroenteritis, involvement of the submucosal or musclular layers is accompanied by intestinal obstruction/stricture, which may be complicated by intestinal perforation [9]. In general, gross findings and micro pathology show mucosal erosion/ulceration or edema and inflammation of eosinophils around the ulcer or perforation site, and absence of the intestinal musculature in the intestinal perforation site, but there are not severe tissue damages [2, 9]. Allergic eosinophilia in the gut can be either superficially oriented, or diffuse, whereas in inflammatory conditions the localization of the eosinophils is always diffuse through the tissue. But, this case presents segmental or partial infiltration of eosinophils in the lesion of intestinal stricture. An allergen-specific lymphocyte response to lactoferrin was markedly increased (7489 counts per minute; stimulation index, 10.11; cutoff index, 3.9). He was finally diagnosed as eosinophilic enteritis with intestinal stricture caused by an allergy to either cow’s milk or breast milk.

Prompt treatment with cow’s milk protein-free formula, including two types of elemental formulas—extensively hydrolyzed and amino acid-based—is effective and leads to clinical remission of eosinophilic enteritis. Our patient experienced an allergy to either cow’s milk or breast milk and developed eosinophilic enteritis with intestinal obstruction. We recommend an amino acid formula as the first choice for feeding in such patients. This particular amino-acid formula consists of dextrins and provides proteinogenic amino acids as a source of nitrogen. There is a small amount of soybean oil but no carnitine or selenium. Clinicians should be aware of the risk of an allergic response to the soybean ingredient in this formula [10]. It is also important to avoid nutritional deficiencies caused by long-term consumption of a single type of formula. It is necessary to monitor for nutritional deficiencies in patients using these formulas over the long term.
